# Building trust in long-term care settings using assistive technology: a systematic review

**DOI:** 10.3389/fresc.2024.1492104

**Published:** 2024-11-22

**Authors:** Kangjie Zheng, Fred Han, Siyu Yang, Nanxin Li

**Affiliations:** School of Systems Design and Intelligent Manufacturing, Southern University of Science and Technology, Shenzhen, China

**Keywords:** trust, long-term care, assistive technology, review, relationship

## Abstract

**Background:**

This review investigates the dynamics of trust between caregivers and care receivers in long-term care settings, where the implementation of assistive technology also becomes chronically crucial. Trust is essential in the care receiver-caregiver relationship as it impacts the effectiveness of care and the care receiver's participation in treatment. Moreover, integrating assistive technology significantly affects the quality of care by increasing care receivers' autonomy and reducing caregivers' workload. Despite its significance, the mechanisms of trust involving assistive technology in long-term care have not been clarified.

**Methods:**

To address this gap, this review systematically analyzed 32 articles published in English since 2,000, sourced from Web of Science, PubMed, Scopus, and Science Direct databases.

**Results:**

The review identified the dynamics of trust in long-term care settings involving assistive technology. Based on this trust dynamics, three critical factors were analyzed: care receiver-related, caregiver-related, and assistive technology-related.

**Discussion:**

The findings provide a detailed understanding of the factors affecting trust in long-term care settings involving assistive technology. These insights contribute to long-term care facility operators making informed decisions regarding technology adoption in care practice and care service strategies, ultimately enhancing trust and the quality of care in long-term care settings.

## 1 Introduction

Trust is the expectation of people's social role behavior in uncertain and risky environments (1). This expectation includes believing the other party is capable, caring, honest, and reliable (2). Additionally, trust is seen as essential in building positive long-term care relationships that benefit care receivers and caregivers (3). Bordin et al. (4) explored the relationship between care receivers and caregivers, highlighting that it evolves from mutual trust. This trust fosters collaboration, enabling both parties to reach shared caregiving objectives. When the bond of caring develops into a more protected recess of inner experience, deeper trust and attachment are needed and cultivated. Existing studies analyzed the trust dynamics between care receivers and caregivers, which are generally based on fundamental theories of trust. According to Mayer et al. (5), the Model of Interpersonal Trust includes trustworthiness characteristics, trust, and risk. Three trustworthiness characteristics, including competence, integrity, and benevolence, must be present before trust can exist. Zhang et al. (6) further divided the trust dynamics into three stages: trust attitudes, intentions, and behaviors developing from initial trust to more vital trust and even evolving into behavioral habits after long-term interaction.

For care receivers, high levels of trust lead to better care receivers' satisfaction with care, reduced anxiety, and increased autonomy and willingness to participate in care. Therefore, in a long-term care relationship, a strong trust relationship can create a positive nursing environment, improve communication, and enhance nursing coordination, which is necessary for a satisfactory and effective caring relationship (7). Additionally, being trusted by caregivers is described as a favorable and verified situation, promoting self-esteem and self-confidence (8).

For caregivers, trust can be conceptualized as the adequate availability of healthcare services and the effective delivery within the care relationship (9). At this level, trust can be assessed based on humanism (listening, accurately understanding, and taking action to address care receiver and family concerns), communication, caregiver knowledge, reliability or competence, and caregiver team functionality (10). Without trust, caregivers may hesitate to provide the necessary care and support, and care receivers may feel vulnerable and apprehensive about receiving assistance (11). However, when trust is established, it can help in creating and sustaining meaningful connections with those they care for, ultimately improving the overall quality of long-term care.

Additionally, assistive technology is crucial in the long-term care relationship between care receivers and caregivers. Ghasemzadeh et al. defined assistive technology as a range of devices, services, and systems that enhance the functional capacities of individuals with disabilities (12). The World Health Organization categorizes assistive technology into six functional domains based on different disabilities: mobility, vision, hearing, communication, cognition, and self-care (13). Examples of assistive technology include a balancing spoon for feeding assistance for Parkinson's patients and an eye tracker for communication assistance for individuals with Amyotrophic Lateral Sclerosis. According to Rani (14), improving a care receiver's ability to live independently can enhance autonomy, self-esteem, self-confidence, and quality of life. For caregivers, assistive technology can simplify and streamline the care process, improve the working environment, and allow more efficient use of time (15). Additionally, assistive devices can foster a stronger and more collaborative long-term care relationship between care receivers and caregivers, as they promote care receiver enthusiasm and confidence in actively participating in their care while reducing caregivers' workload (16).

However, some articles reported that using assistive technology may also bring challenges. Some care receivers find the devices complicated, troublesome, and embarrassing or feel that they do not meet their expectations and fail to gain their trust (17). For example, a study on exoskeleton technology found that 33.8% of care receivers experienced unintended misuse when using exoskeleton robots, and 50.8% of care receivers encountered accidental device activation, which decreased their trust in assistive technology (18). Trust is essential for users to embrace assistive technology. It's crucial to comprehensively analyze the role of trust in the long-term care process, as it directly impacts the quality of care provided through assistive devices.

Therefore, if the care receiver's physical condition gradually deteriorates in long-term care, the demand for assistive technology from both care receivers and caregivers will increase, making its use the norm. Moreover, as the care receiver's physical condition becomes more fragile, they become more sensitive to potential care risks, affecting the trust between care receivers and caregivers. Therefore, examining the dynamics of trust in long-term care settings involving assistive technology is essential.

Assistive technology plays a significant role in care relationships and should not be overlooked in discussions about trust in long-term care. Ignoring the context of assistive technology use can lead to a misunderstanding of the power dynamics between care receivers and caregivers (19). For instance, care receivers using wheelchairs can move independently, leading to increased confidence and a decreased need for caregiver assistance, ultimately giving care receivers more independence in decision-making. However, current research often fails to consider the practical impact of assistive technology on trust in long-term care (20). This review aims to address this gap by considering the context of assistive technology when discussing trust in long-term care.

## 2 Methods

This review followed a structured approach to retrieve and screen articles by the PRISMA guidelines to address the question (21): What are the trust dynamics between care receivers and caregivers when using assistive technologies in long-term care settings? This review explores the care receiver's trust in assistive technology, the caregiver's trust in assistive technology, and the mutual trust between care receivers and caregivers involving assistive technology. By analyzing these relationships, the review aims to clarify the critical role of assistive technology in trust dynamics in long-term care settings and the factors influencing them. The findings of this review could potentially contribute to a better understanding of trust in long-term care through assistive technology, thereby enhancing the effectiveness of long-term care.

### 2.1 Search strategy

The search process followed a systematic approach, using clear procedures and reflective processes recommended by Harcourt and Rumsey (22) to identify and review articles. This approach helped to locate all relevant literature that met the predetermined inclusion criteria and avoid missing critical studies or mistakenly including out-of-scope studies, which could bias the analysis. The search included four concepts: long-term care, trust, assistive technology, and assistive devices. The words were entered individually into designated databases related to the research topic to identify synonyms, phrases, alternative terms, related terms, plurals, and word spelling variations (23). This review involved both professional and informal caregivers, including family members. It focused on care receivers who require long-term assistance and rely on caregivers for daily activities, highlighting their greater need for assistive technology. Long-term care in this context encompassed home care and institutional settings like nursing homes. Four databases were used: Web of Science, PubMed, Scopus, and Science Direct. Boolean logic operators like “AND” and “OR” were used to limit the search. The search focused on peer-reviewed evidence syntheses published after 2000. This timeline was determined based on trends noted in two reviews on assistive technologies in long-term care that indicated that most papers were published after 2000 (24, 25). The language of the study had no restrictions during the search. The initial search was conducted in May 2024, followed by an updated search in June 2024 using the same strategy.

### 2.2 Eligibility criteria

#### 2.2.1 Inclusion criteria

• Topic of study:
  (1) were set in long-term care settings.
  (2) discussed relationships and/or trust between care receivers and caregivers.
  (3) included assistive technology that allowed for interaction or information sharing between care receivers and caregivers.
• Type of scientific material to analyze: Primary studies (quantitative, qualitative, and mixed methods) were included as they could provide insight into the phenomenon under study.
• Full text available. Only full-text and conference articles.

#### 2.2.2 Exclusion criteria

• Articles that did not address persons in long-term care.
• Articles that did not include assistive technology in long-term care.
• Articles that did not discuss trust in long-term care while using assistive technology.
• Articles exclusively discussing care receivers aged below 18 years were excluded, as the review focused on adults receiving long-term care services.

### 2.3 Study selection and evaluation

The text screening process was divided into two stages: (1) title and abstract and (2) full text.

(1) Title and abstract screening: A panel of three members reviewed the articles' titles and abstracts in two rounds to ensure they met the inclusion criteria. All three members had a background in assistive technology research. Each member screened a random sample of 100 titles and abstracts, marking the results as “include” and “exclude.” If the panel members could not judge whether to include an article, it was marked for “further discussion.” After the first screening round, a meeting was held to discuss the screening results. For the articles that could not be judged, the review panel discussed to reach a consensus on whether these studies should be included or excluded from the screening.

Study evaluation: After the second screening round, the screening results were checked for consistency. Cohen's Kappa values were calculated between pairs of panel members (for example, Panel 1—Panel 2; Panel 2—Panel 3; Panel 1—Panel 3), resulting in Kappa values ranging from 0.66 to 0.854, indicating moderate to high agreement (26).

(2) Full-text screening: At the full-text screening stage, a single-reviewer approach was considered sufficient due to a clear understanding of the inclusion and exclusion criteria established by the panel members and due to time and resource constraints. Additionally, this review included studies that met the inclusion criteria without a formal quality assessment due to the low search yield. This approach allowed us to compile a wider array of data, providing broader insights into the research conclusions.

### 2.4 Data extraction and synthesis

The research panel conducted data extraction and analysis. Three panel members extracted data using a form developed specifically for this review. The form was piloted in three articles and included information: type of assistive technology, description of care receivers, description of caregivers, and data collection method. Assistive technologies were classified according to SCHOLZ's and Mishra's description of assistive technologies (see Table 1) (27, 28). All studies identified in the search were thoroughly read, and initial codes were given to subsets of the data following the approach proposed by Thomas and Harden (29). A coding strategy involving descriptive and evaluative coding was employed. Descriptive coding involves assigning codes that best describe the flow of data. In contrast, evaluative coding involves assigning codes based on judgments about the presence of concepts, as defined by Miles et al. (30).

**Table 1:** Classification of assistive technologies.

Category	Description
Mobility	Mobility assistive technologies could be used for motor disabilities, or mobility impairments, affect the upper and/or lower limbs, such as wheelchairs, lower-limb prostheses, and walkers.
Vision	Vision assistive technologies could be applied to visual impairment such as cane and bionic eyes.
Hearing	Hearing assistive technologies are suitable for hearing loss, or deafness, such as hearing aids.
Communication	Communication assistive technologies focus on alternative and augmentative communication. It can be used for speech disorders caused by diseases or remote communication of disease information.
Cognition	Cognition assistive technologies are applied to symptoms that affect cognitive ability and intelligence, such as Alzheimer's disease, traumatic brain injury, assisted comprehension problems, reading, and verbal comprehension. For example, social assistance robots.
Self-care	Self-care assistive technologies can be used for washing oneself, caring for body parts, toileting, dressing, eating, drinking and caring for healthy. For example, bathing aids, feeding aids and sphygmomanometer.

Thematic analysis was conducted to identify the impact of six types of assistive technology on the care receiver-caregiver relationship, trust, and any influencing factors. Two panel members independently coded the data from each article on influencing factors. This involved highlighting and marking relevant sections from the extracted data in a Word document. Following this, two panel members met to discuss and merge the developed codes into a single document. Subsequently, the first member then analyzed the codes and identified three major categories: care receiver-related, caregiver-related, and technology-related. The second member reviewed these categories to ensure they accurately represented the data extracted from the articles.

## 3 Result

**Figure 1:**
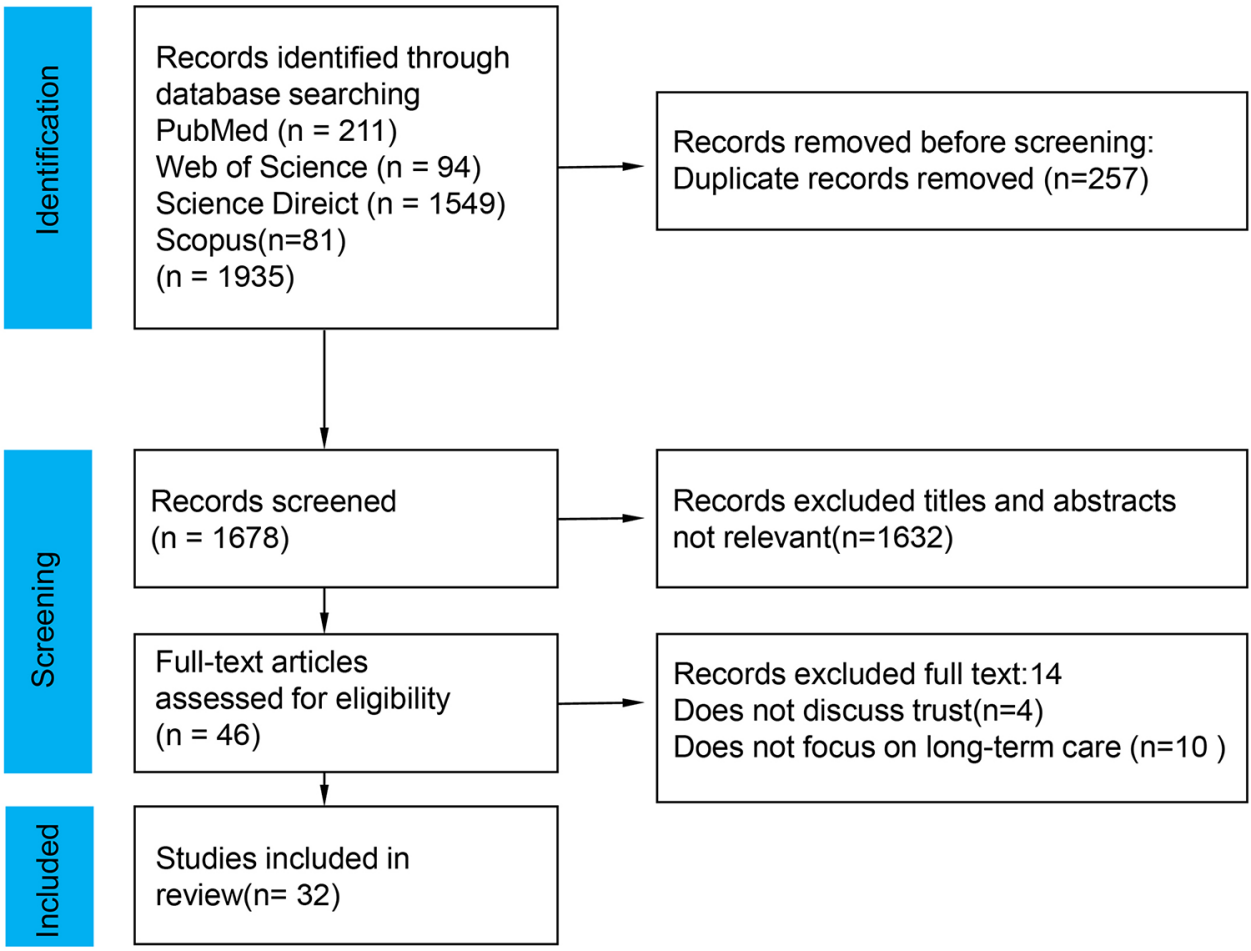
Prisma flow diagram.

The database search retrieved 1,935 references, and 257 duplicate articles were removed. 1,632 articles were excluded through the title and abstract screening stage. The remaining 46 articles were reviewed in full text. Out of these, 14 articles were discarded because they did not meet the inclusion criteria (see Figure 1).

### 3.1 Overview of articles

**Figure 2:**
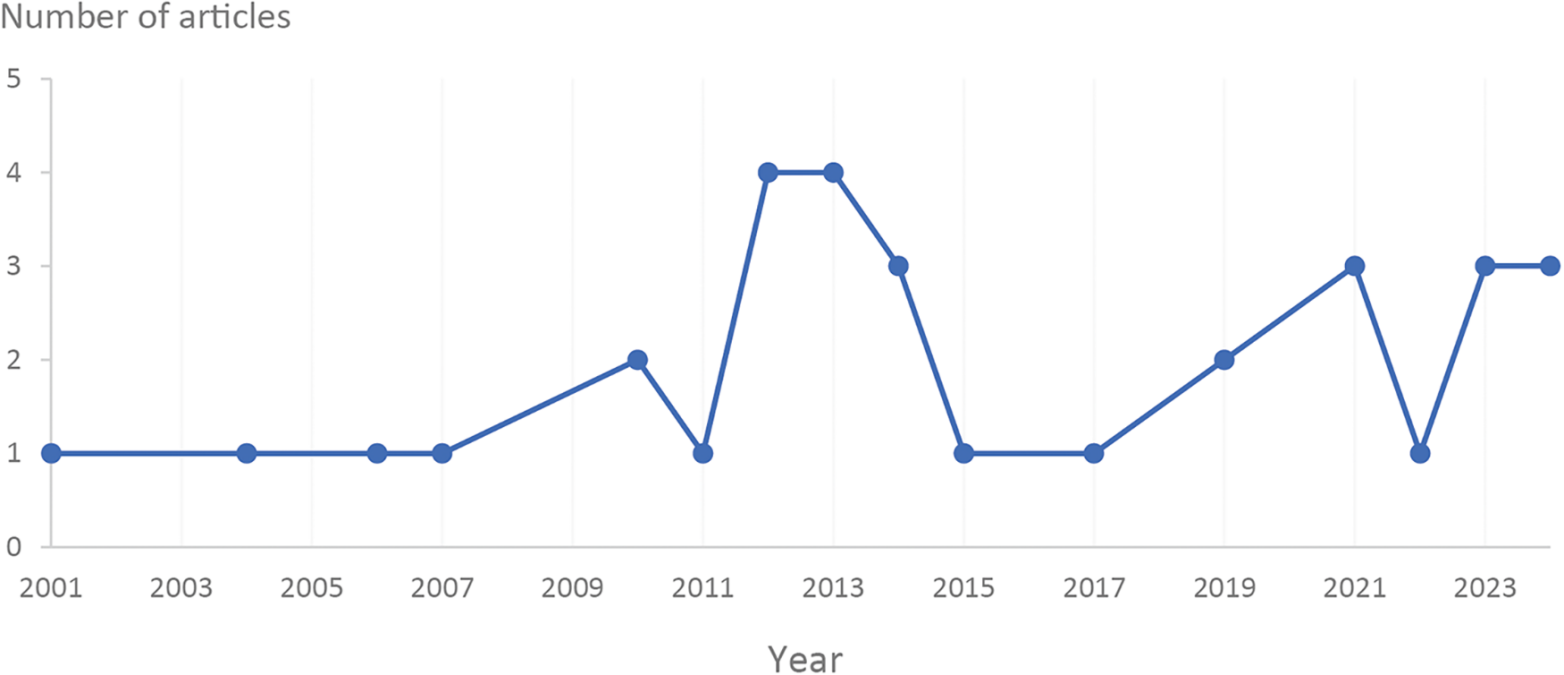
Number of articles by year.

A total of 32 articles were included in the screening process. Most articles were published from 2012 onwards, with the highest number of articles in 2012 and 2013 (see Figure 2). Among the six domains of assistive technology, self-care assistive technology, such as electronic health self-management tools, was the most frequently discussed (*n* = 13). This was followed by articles discussing communication assistive technology (*n* = 6), such as socially assistive robots, and mobility assistive technology (*n* = 6), including wheelchairs and walkers.

Most of the articles discussed the physical conditions of the care receivers. These conditions included chronic kidney disease (*n* = 1), cognitive impairment and Alzheimer's disease (*n* = 5), stroke and lower limb disability (*n* = 6), hearing impairment (*n* = 1), chronic obstructive pulmonary disease (*n* = 3), visual impairment (*n* = 2), and diabetes complicated with multiple physical conditions (*n* = 10).

Some of the articles described the situation of caregivers, which included informal carers (*n* = 6), such as family members and friends of care receivers; professionals (*n* = 6) like nurses, therapists, and general practitioners; professional caregivers working in facilities, and care receivers' family caregivers (*n* = 3). Table 2 outlines the characteristics of the articles that are included.

**Table 2:** Description of articles.

Authors	Type of assistive technology	Description of care receivers	Description of providers	Data collection
Murphy et al. (31)	Health technologies (Self-care)	Older adults living with multiple chronic health conditions, such as Diabetes and Coronary Heart Disease	Nurses, physiotherapists, occupational therapists	Semi-structured individual interviews and focus groups
Donald et al. (32)	Electronic health self-management tool (Self-care)	Chronic kidney disease	Primary care physicians, allied health	Semi-structured interviews
Mehrabianet al. (33)	Home telecare system (Cognition)	Cognitively impaired and Alzheimer's disease	Care receiver's family	Semi-structured interviews
Ziefle et al. (34)	Video-based monitoring systems (Communication)	Older adults in long-term care	Not described	Scenario-based questionnaire
Copolillo, Albert E. (35)	Mobility devices (Mobility)	Lower limb disables	Nurses	Focus groups and individual narrative interviews
Barker et al. (36)	Wheelchair (Mobility)	Senior stroke survivors	Not described	Semi-structured, in-depth interviews
Lindqvist et al. (37)	Assistive technology for cognitive support in everyday (Cognition)	Alzheimer's disease	Care receiver's family	Semi-structured interviews
Gramstad,et al. (38)	Wheeled walker, shower chair, Bidet Lift chair (Mobility)	Difficulties with mobility	Care receiver's family	Interviews
Southall et al. (39)	Hearing assistance technology (Hearing)	Hearing impaired persons	Care receiver's families and friends	Interviews
Pettersson et al. (40)	Assistive devices for Mobility, personal care and housekeeping (Mobility)	Post-stroke individuals	Spouses and home help staff/personal assistant	Conversational interviews
Huniche et al. (41)	Self-monitoring (Self-care)	Chronic obstructive pulmonary disease	Spouses and other family members	Semi-structured interviews
Lu et al. (42)	Home telehealth (Communication)	Primary diagnosis of hypertension, diabetes or both	Nurses	Focus group meeting and individual interviews
Fairbrother et al. (43)	Telemonitoring (Communication)	Chronic obstructive pulmonary disease	Healthcare professionals and managers	Semi-structured interviews
Chang et al. (44)	Home Telehealth Technology (Communication)	Diabetes	Family members, doctors and nurses	Interviews
Fairbrother, Peter, et al. (45)	Telemonitoring (Communication)	Chronic obstructive pulmonary disease	Professionals such as nurses and general practitioners	Semi-structured interviews
Conradie et al. (46)	Tactile systems (Vision)	Visually impaired	Not described	Interviews
Wilkowska et al. (47)	Medical Assistive (Self-care)	Chronic disease such as cardiovascular diseases, diabetes mellitus, asthma	Not described	Focus group and questionnaire
Harrefors et al. (48)	Digital Photo Diary (Cognition)	Mild Dementia	Professional caregivers	Questionnaire
Balasubramanian et al. (16)	Voice activated device ’smart speaker’ (Self-care)	Diabetes and the other on a range of long-term health conditions such as multiple sclerosis, dementia, depression	Informal carers	Focus groups
Sun et al. (49)	Personal health devices (Self-care)	Care receiver suffering from different chronic diseases, including hypertension, hyperthyroidism, arthritis diabetes, coronary disease, bronchitis	Not described	Questionnaire
Materia et al. (50)	Wearable health sensors (Self-care)	Chronic health conditions such as AIDS, Arthritis, Asthma, Diabetes and Emphysema	Not described	Questionnaire
Wang et al. (51)	AI-assisted diagnosis in intelligent Healthcare (Self-care)	Care receivers suffering from different chronic diseases, including hypertension, diabetes, chronic obstructive pulmonary disease	Doctors	Questionnaire
Tan et al. (52)	Assistive robots (Self-care)	Chronic conditions, such as diabetes, hypertension	Informal caregiver such as family members	Questionnaire
Farina et al. (53)	Wearing activity monitors (Self-care)	Older adults with dementia	Carer in community	Qualitative interviews
Milallos et al. (54)	Smart cane (Vision)	Blind or visually impaired	Not described	Online interview
Ventura et al. (55)	Artificial intelligence system named MAIA would aim to interpret users’ intentions and translate them into actions performed by assistive devices (Mobility)	Post-stroke	Not described	Socio-demographic questionnaire and interview
Wilkowska et al. (56)	Medical device – a smart textile (Self-care)	Chronic disease	Not described	Questionnaire
Lingg et al. (57)	Autonomous wheelchair (Mobility)	People with mobility impairments	Not described	Questionnaire
Pino et al. (58)	Socially assistive robots (Cognition)	Persons with Mild Cognitive Impairment	Informal caregivers	Questionnaire
Heerink et al. (59)	Socially assistive robots (Communication)	Chronic disease	Not described	Questionnaire
Skymne et al. (60)	Assistive device (Self-care)	Older adults with multiple health problems affecting daily activities	Not described	Group discussion
Otten et al. (61)	Assistive device (Self-care)	Chronic diseases	Not described	Semi-structured interviews

### 3.2 Care receiver-caregiver trust involving assistive technology

**Figure 3:**
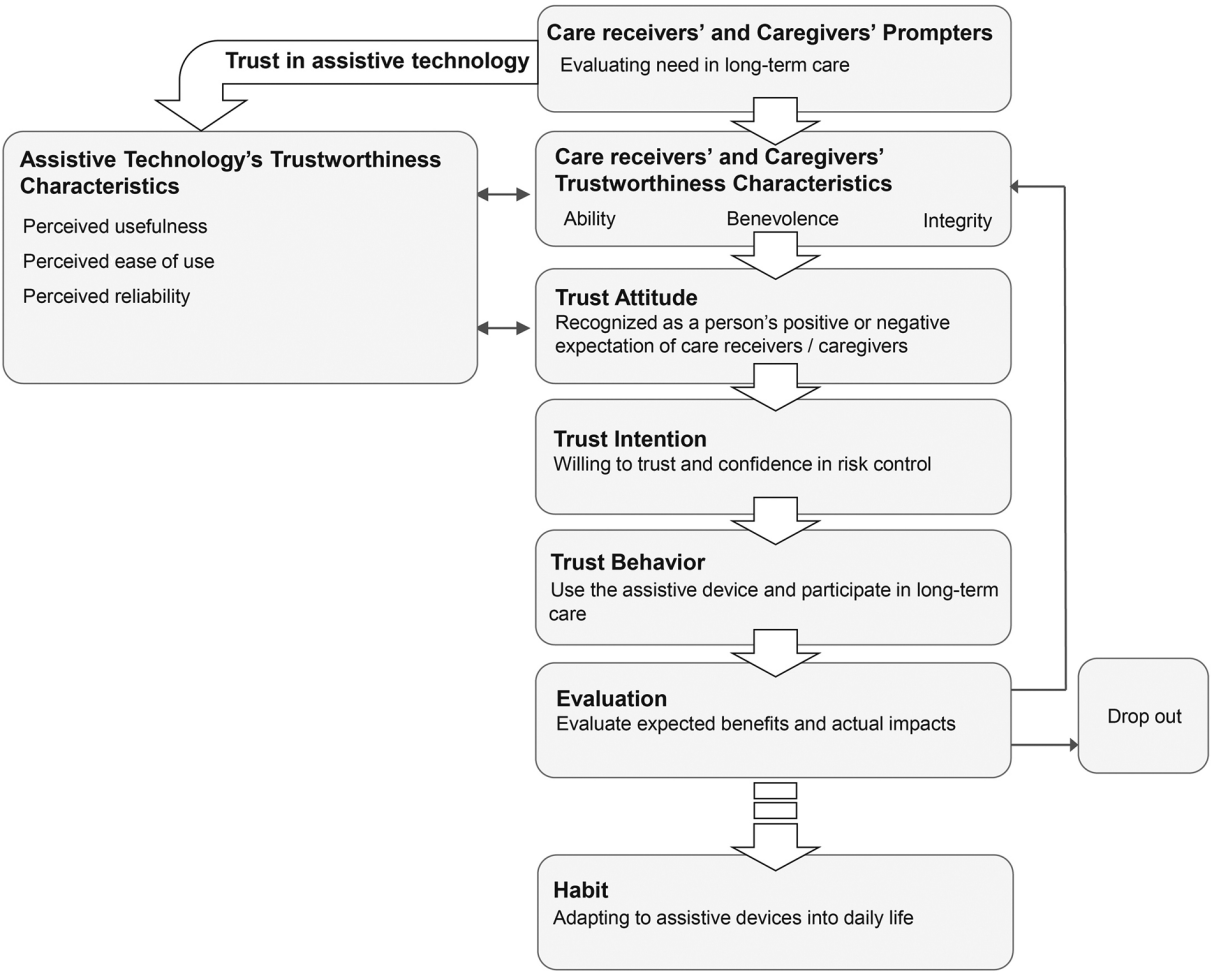
Care receiver-caregiver trust involving assistive technology.

Care receiver-caregiver trust involving assistive technology includes care receiver and caregiver trust in assistive technology, and care receiver-caregiver trust through assistive technology.16 articles discussed care receiver trust in assistive technology (31, 32, 34, 37, 42, 44, 45, 47–49, 51, 52, 55, 58–60), while 7 articles discussed caregiver trust in assistive technology (32, 43, 48, 51, 52, 55, 58). More articles linked care receiver and caregiver trust in assistive technology with the acceptance process. They referred to theories like the Theory of Acceptance and Use of Technology (UTAUT) (49, 51, 52, 56, 58). However, only a few articles examined care receiver and caregiver trust in assistive technology from the perspective of interpersonal trust (40, 55). According to Pettersson et al. (40), risks from the perspective of interpersonal trust must be taken seriously when using assistive technology, as risk factors such as safety, privacy, and dignity are crucial for care receivers in long-term care. Therefore, this review starts with the perspective of interpersonal trust and combines it with the descriptions of the technology acceptance process in most articles, proposing a process for care receiver-caregiver trust involving assistive technology. This process starts with prompters, contacting trustworthiness characteristics of assistive technology, care receivers, and caregivers, generating trust attitude, trust intention, and trust behavior, and finally, evaluating assistive technology and slowly evolving it into life habits (see Figure 3).

#### 3.2.1 Prompters

Care receivers evaluate their long-term care needs throughout this process. These needs are related to their physical limitations when performing daily activities (31, 49). For example, those with lower limb disabilities often require assistive technology to use the toilet independently. Consequently, limitations in long-term care prompt care receivers to use assistive technology (55). During this phase, care receivers also develop expectations regarding how assistive technology can help them overcome challenges posed by their physical condition (51).

Caregivers assess the complexity of nursing tasks in this process. The complexity of tasks will trigger the caregiver's interest in assistive technology (48, 52). For example, Pino et al. (58) reported that caregivers who care for care receivers with cognitive impairment have a high demand for social assistive robots, as communicating with care receivers with cognitive impairment can be challenging.

#### 3.2.2 Care receivers' and caregivers' trustworthiness characteristics

These characteristics encompass ability, benevolence, and integrity. For care receivers, these characteristics reflect their capacity to perform daily activities and manage health conditions independently, willingness to communicate and collaborate with caregivers, and consistency in actions (42, 45, 50). On the other hand, caregivers demonstrate trustworthiness through their ability to obtain the necessary nursing knowledge and skills, kindness in communication, and dedication to upholding the dignity and autonomy of care receivers (40, 48, 60). However, in long-term care settings, care receivers' trustworthiness characteristics change due to physical conditions; accordingly, their requirements for caregivers' trustworthiness characteristics also change (43).

#### 3.2.3 Assistive technology's trustworthiness characteristics

Both care receivers and caregivers assess the usefulness, reliability, and usefulness of assistive technologies (49, 51, 52, 56, 58). Huniche et al. (41) identified several factors influencing care receivers' perceptions of the technology's trustworthiness, including referrals from family, friends, and professional caregivers. Some articles indicate a link between the trustworthiness characteristics of assistive technologies and the trust perceptions of care receivers and caregivers (49, 55, 58). For example, Pino et al. (58) found that when family members of care receivers with cognitive impairments trusted social assistive robots, they felt more assured that their loved ones could engage socially in the community.

#### 3.2.4 Trust attitude

Care receivers and caregivers evaluate the advantages and disadvantages of assistive technology. Tan et al. (52) outlined the evaluation process, which includes assessments of financial risk, performance risk, perceived usefulness, ease of use, and overall well-being, ultimately shaping the perceived value of assistive technology. Caregivers must consider their convenience and the well-being of those they care for when evaluating assistive technology (41). Furthermore, the reciprocal influence between the trustworthiness of assistive technology and caregivers' trust attitudes was mentioned in the collected articles (34, 43, 55). For example, Ventura et al. (56 noted that caregivers feel intelligent wheelchairs reduce risks for care receivers with lower limb disabilities when crossing roads, making them feel more at ease using such devices. Consequently, assistive technology can empower both care receivers and caregivers, mitigate risks in caregiving, and foster trust between them.

#### 3.2.5 Trust intention

Care receivers' and caregivers' willingness to trust assistive technology. For care receivers, this involves confidence in the reliability of such technology and believing that any unexpected risks will be effectively handled (55). Caregivers, in turn, feel assured in their capacity to mitigate the potential risks associated with using assistive technology. For example, when using remote health monitoring systems, caregivers should remain attentive to safety concerns and potential privacy violations that may impact care receivers (42). Consequently, trust intention emerges from carefully evaluating the associated risks and benefits.

#### 3.2.6 Trust behavior

Care receivers and caregivers engage with assistive technology while participating in long-term care (52, 58).

#### 3.2.7 Evaluation

Care receivers and caregivers assess the actual outcomes of assistive technology against anticipated benefits. For care receivers, these evaluation results significantly impact their future interactions with assistive technology, affecting their degree of trust and attitude. If the actual outcomes are disappointingly low compared to expectations, the care receiver might discontinue using the assistive technology (32, 44, 51).

#### 3.2.8 Habit

Integrating assistive technology into everyday life. However, some care receivers are worried about the long-term reliance on assistive technology, fearing it could foster dependence and cause social stigma (60). Moreover, this habit might be temporary for care receivers, whose needs often change as their physical condition progresses. When their condition changes, they must re-establish trust in assistive technology to meet their evolving care requirements (45). For example, as stroke patients experience declining lower limb mobility, they are prompted to reassess their trust in the daily crutches they use and determine if they should consider alternative mobility assistive devices.

### 3.3 Impact factors of trust in long-term care

From the above description, it is apparent that although the trust process of caregivers and care receivers in assistive technology is similar, the factors affecting their trust in assistive technology differ because of the various roles they play in the long-term care relationship. Additionally, assistive technology plays a dual role in establishing trust with care receivers and caregivers. This review identifies the influencing factors of care receivers, caregivers, and assistive technology. It categorizes the number of articles related to each influencing factor according to the type of assistive technology.

#### 3.3.1 Care receiver-related factors

18 articles mentioned care receiver-related factors (31, 34, 37, 40–47, 49, 50, 54, 55, 58, 60). Among these factors, physical condition and experience in assistive technology were mentioned the most, with six articles, respectively, followed by social willingness and financial conditions, with four articles. Additionally, demographics, experience in care receiver-caregiver and propensity for independence were mentioned in two articles, as shown in Table 3.

**Table 3:** Care receiver-related factors.

Care receiver-related factors	Number of article (classified by the type of assistive technology)					
	Mobility	Vision	Hearing	Communication	Cognition	Self-care
Demographics: gender, age					*	*
Physical condition	*			*		****
Experience in care receiver-caregiver relationship	*			*		
Experience in assistive technology		*		**		***
Social willingness	*			**		*
Propensity in independence	*			*		
Financial conditions: Covering the costs of care and assistive technology				**	*	*

Factors related to physical condition include the current physical state and expectations regarding it. Care receivers are more apprehensive about assistive technology's potential adverse effects on their physical health and overall well-being. This heightened concern may be due to the fragile physical function of care receivers, as they require assistance but have a lower tolerance for the potential risks associated with assistive technology (50). Additionally, the reasonable expectations of care receivers regarding their physical condition impact their trust in caregivers and assistive technology, which was reported in articles discussing self-care assistive technology (31, 41). According to Murphy et al. (31), if care receivers have unrealistic expectations for their physical condition, they may experience anxiety during the prolonged recovery process and ultimately lose trust in both caregivers and assistive technology.

Regarding experience with assistive technology and the care receiver-caregiver relationship, many care receivers encountered incorrect operations and technical failures while using assistive technology. These experiences made them feel embarrassed, stressed, and worried about the reliability of assistive technology (46). As a result, their willingness to use assistive technology decreased (44). Additionally, the experience of interacting with caregivers influences care receivers' trust in caregivers. This, in turn, can lead to care receivers being hesitant to seek help and feeling afraid of disturbing others (40, 44).

Regarding social willingness, articles discussing mobility, communication, and self-care assistive technology have mentioned how these technologies can disrupt interpersonal interactions (31, 42, 43, 55). Care receivers desiring social interaction are concerned that using assistive technology may hinder their ability to connect with others (31, 42). However, maintaining interpersonal relationships with caregivers can improve their satisfaction with long-term care (43). For instance, Patterson et al. (32) found that care receivers were denied help by caregivers, causing the care receivers to feel ashamed about relying on others. Furthermore, care receivers with a propensity for independence were more inclined to solve problems encountered in care alone rather than relying on the working hours and help opportunities provided by caregivers (40, 45).

In terms of financial conditions, care receivers have to coexist with their condition, requiring long-term and complex care. For example, articles discussing multiple sclerosis, dementia, and depression mention the high long-term care and assistive technology costs as the disease progresses, creating barriers for care receivers to receive the care they need (16, 33, 42, 44).

The discussion on demographics included factors such as gender and age. According to Wilkowska et al. (56), middle-aged care receivers have the lowest confidence in the reliability of assistive technology compared to elderly and young care receivers. Young care receivers value privacy the most when using assistive technology and often hide this usage from others. Additionally, the trust of male care receivers is significantly influenced by the perceived availability of assistive technology, while the factors female care receivers consider are more complex.

#### 3.3.2 Caregiver-related factors

16 articles mentioned caregiver-related factors (16, 31, 35, 39, 42–45, 48, 49, 51, 52, 54, 55, 58, 60). Among these factors, experience in assistive technology was mentioned the most, with six articles, followed by communication skills and collaboration, with five articles. Additionally, competency and knowledge were mentioned in four articles, and experience in care receiver-caregiver and working burden were mentioned in three and two articles, respectively (see Table 4).

**Table 4:** Caregiver-related factors.

Caregiver-related factors	Number of article (classified by the type of assistive technology)					
	Mobility	Vision	Hearing	Communication	Cognition	Self-care
Competency and knowledge				*	*	**
Working burden					*	*
Experience in care receiver-caregiver relationship	*					**
Experience in assistive technology	*				*	****
Communicate skill				**	*	**
Collaboration	*			**		**

Caregivers' experience in assistive technology plays a key role in the quality of care, as caregivers have the final say in deciding how to use assistive technology (60). Articles discussing self-care assistive technology mention caregivers refusing to use digital assistive devices for care receivers because they believed the complex operation procedures were unsuitable (61).

Regarding communication skills and collaboration, caregivers' positive and supportive communication skills can help care receivers gain a sense of security, recognition, and self-esteem in care. For example, Lu et al. (42) reported that remote health monitoring systems might weaken face-to-face social interaction, which will increase care receivers' anxiety and panic when they encounter problems. Additionally, the collaboration mode between caregivers and care receivers without assistive technology differs in that it involves assistive technology. For example, for care receivers using wheelchairs, caregivers need to establish a new cooperative relationship with care receivers, take on the roles of cleaning wheelchairs, and provide continuous support to care receivers when they cross the road to enhance their sense of security (40). Therefore, an excellent collaborative model can promote care receivers to obtain high-quality care.

Regarding competency and knowledge, on the one hand, the caregiver's familiarity with nursing expertise affects their initial trust in assistive technology, as nursing expertise will support them in controlling the risks of using assistive technology (48, 51). On the other hand, the professional ability of the caregiver was regarded as a guarantee for care receivers to use assistive technology. According to Lu et al. (42), care receivers believe that the professional guidance of the caregiver can enable them to use assistive technology better. Additionally, experience in the care receiver-caregiver relationship and the working burden of caring tasks affect the caregiver's motivation to use assistive technology (41).

#### 3.3.3 Assistive technology-related factors

Sixteen articles mentioned assistive technology-related factors (34, 37, 40–42, 44–47, 49, 52, 54, 55, 58, 60, 61). Among these factors, perceived usefulness and perceived reliability were mentioned the most, with eight articles, respectively, followed by perceived ease of learning and social support, with four articles. Additionally, morality and cost were mentioned in three articles, respectively (see Table 5).

**Table 5:** Assistive technology-related factors.

Assistive technology-related factors	Number of article (classified by the type of assistive technology)					
	Mobility	Vision	Hearing	Communication	Cognition	Self-care
Perceived usefulness	*	*	*	**		***
Perceived ease of learning	*			*		**
Perceived reliability		*	*	***	*	**
Cost				**		*
Morality				*		**
Social support	*			*		**

Perceived usefulness is the factor that receives the most attention from care receivers and caregivers because it affects their expectations of the functions of assistive technology. For care receivers and caregivers in long-term care settings, perceived usefulness means providing care receivers with short-term safety and comfort and providing long-term functional support to adapt to the progression of the disease. For example, Skymne et al. (60) reported that some care receivers expected to improve their physical function in the long-term care process because they were worried about becoming dependent on assistive technology and could not live everyday life without it.

Perceived reliability impacts care receivers' continued use of assistive technology. Especially when assistive technology gains the initial trust of care receivers and caregivers, the most important factor affecting their continued use of assistive technology is that the technology can operate reliably and stably (44).

Care receivers and caregivers are more likely to trust easy-to-use assistive technology. On the contrary, when caregivers and care receivers make mistakes in complex operation processes and try to use them repeatedly, their psychological pressure will increase, and they will quickly lose trust in assistive technology, eventually leading to its abandonment (49).

Regarding social support, assistive technology must be provided to care receivers. On the contrary, assistive technology that breaks interpersonal interaction will make care receivers helpless. For example, Skymne et al. (60) reported that some caregivers believed that care receivers no longer needed help after using assistive technology, which caused care receivers to feel helpless and embarrassed when encountering difficulties, reducing their recognition of assistive technology and caregivers.

Morality involves the privacy, dignity, and positive emotional support of care receivers (49). According to Ziefle et al. (34), only by thoroughly ensuring that assistive technology respects privacy restrictions, is unobtrusive, invisible, and provides tangible support can a reasonable level of acceptance be achieved.

## 4 Discussion

Compared with the trust dynamic between care receiver and caregiver without assistive technology, care receiver-caregiver trust involving assistive technology includes the process of trust in assistive technology, and trust in assistive technology impacts care receiver-caregiver trust by interacting with their trustworthiness characteristics and trust attitude. According to existing research discussing care receiver-caregiver trust, trustworthiness characteristics are necessary to promote trust between care receivers and caregivers. For example, Mikesell et al. (62) reported that care receivers trust caring offered by caregivers with competence, knowledge, and integrity. However, this conclusion cannot fully cover some actual long-term care scenarios of using assistive technology, such as care receivers accepting care from family members with limited nursing knowledge but knowing how to use assistive technology. This review summarizes the mechanism of care receiver-caregiver trust involving assistive technology, which can explain the trust dynamics between care receivers and caregivers when using assistive technology.

On the one hand, trust in assistive technology interacts with care receivers' and caregivers' trustworthiness characteristics. Trust in assistive technology empowers care receivers' and caregivers' trustworthiness characteristics. Sun et al. (49) reported that personal health assistive technology can promote care receivers' recognition of caregivers' caring ability by strengthening communication between care receivers and caregivers. Care receivers' and caregivers' trustworthiness characteristics can also support trust in assistive technology. According to Otten et al. (61), care receiver's trust in assistive technology is built on not only factors like function, empathy, transparency, and communication but also the professional competence of caregivers who recommend the assistive technology, especially when care receivers use assistive technology for the first time. However, the critical effect between trust in assistive technology and care receivers' and caregivers' trustworthiness characteristics may also be harmful. For example, the difficulty in operating social assistive robots will put pressure on care receivers with dementia and cause them to resist caregivers' work (58).

On the other hand, trust in assistive technology interacts with care receivers' and caregivers' trust attitudes. Assistive technology affects care receivers' and caregivers' trust attitude through risk management. For example, Pettersson et al. (40) reported that mobility assistive devices can reduce care receivers’ risk of falling, which leads to the care receiver's family agreeing care receivers to leave alone and even take care of household chores while using mobility assistive devices. However, assistive technology also brings potential risks. For example, Fairbrother et al. (43) argued that the use of telehealth detectors may cause care receivers' loss of patience and confidence in the caregivers' caring, as this assistive technology brings potential risks resulting in care receivers overly focusing on their physical data, and getting anxiety about the physical condition.

Therefore, when using assistive technology, it is necessary to develop personalized guidelines for using assistive technology based on the characteristics of care receivers and caregivers. The guidelines for using assistive technology involve guiding the selection of assistive technology and standardizing caregivers' introducing, teaching, and using assistive technology for care receivers. Standardized guidelines will promote the trust of care receivers and caregivers, as they can control the risks of assistive technology and give full play to its advantages.

Assistive technology-related factors play a critical role in the relationship between care receiver-related factors and caregiver-related factors, which promotes coordination between influencing factors and ultimately impacts the trust dynamic. This review found that assistive technology's perceived usefulness and reliability coordinate the care receiver's physical condition and the caregiver's communication skills and collaboration. Firstly, most articles mentioned the care receiver's physical condition because the deterioration of physical condition leads to an increase in care needs, which is the core of long-term care. For example, as the physical condition of care receivers with chronic obstructive pulmonary disease changes, on the one hand, they need to adjust their expectations of their physical condition. On the other hand, they need to rely on more care, such as using a ventilator to maintain survival (63). Therefore, the increase in care receivers' care needs and the intervention of assistive technology pose new challenges to caregivers' work. Secondly, caregivers' communication skills and collaboration are important factors in adapting to care receivers' physical changes. When assistive technology is involved in long-term care, caregivers must adjust their roles and provide care receivers with operational assistance and emotional support. For example, when care receivers use digital personal health monitoring devices, caregivers need to assist care receivers in understanding their health data and establishing correct expectations about their physical condition. Otherwise, incorrect cognition and operation of assistive technology will cause the care receiver to feel anxious and have low self-esteem regarding their physical condition (64). Finally, assistive technology's perceived usefulness and reliability promote cooperation between care receivers and caregivers. The perceived usefulness and reliability of assistive technology encourage care receivers to participate in care activities more actively, as assistive technology provides care receivers with the expectation of improving their physical function. Additionally, assistive technology guides caregivers to participate in care efficiently and gain the recognition of care receivers. For example, digital personal health monitoring devices provide diet planning for diabetic care receivers, guide caregivers to prepare diets that suit the care receiver's physical condition, and promote care receivers' recognition and trust in the professional ability of caregivers (65).

Implications for using assistive technology in long-term care setting. (1) Promote interpersonal interaction and social support through assistive technology. For care receivers, using assistive technology can promote their autonomy, but it does not mean they can maintain complete independence without interacting with the caregiver. Assistive technology can complement, rather than replace, caregivers by offering professional advice and strengthening care-receiver-caregiver interactions. For example, using an AI health management system does not mean that care receivers do not need the help of caregivers. Instead, it provides more professional advice through AI to promote care receivers' recognition of caregivers and strengthen the interaction between care receivers and caregivers (66). (2) Focus on the care receiver's physical condition and pay attention to the care receiver's emotional support. In everyday life, care receivers may experience multiple health issues. Those with diverse medical conditions often have mixed feelings regarding assistive technology, as they see its potential to create opportunities and offer a sense of security while being aware of its limitations and concerns (60). On the one hand, assistive technology's customizable and adaptable nature supports its capacity to address personalized needs and eliminate barriers caused by various physical conditions. Conversely, assisting care receivers in understanding their multiple health issues and providing positive emotional support can help them adjust their expectations, thus increasing their trust in assistive technology. For example, Huniche et al. (43) reported that caregivers need to provide positive emotional support for care receivers when using health-detecting technology. Physical data health from assistive technology is not neutral to care receivers but feedback on their physical status, which they are mostly concerned with. Therefore, it is necessary to pay attention to assistive technology's positive or negative emotional value. (3) From care receiver dependence on assistance to collaborative assistance. Care receiver-centered care tends to heighten care receivers' dependence on their caregivers, which in turn worsens their social stigma. When care receivers must rely on caregivers for daily tasks, they often experience diminished decision-making power, leading to a decline in self-worth and increased social labelling as disabled. The resulting social stigma and dependency pressures can make care receivers resistant to assistive technology (67). On the other hand, collaborative assistance promotes the active participation of care receivers in their care, bolstered by assistive technology, which can enhance their sense of self-worth and alleviate caregiver burdens. Research has indicated that effective collaborative care correlates with improved management of health indicators and greater treatment success (68). However, it's essential to recognize that not every care receiver wishes to engage actively in care decisions; some may prioritize pleasing their caregivers over participating in these decisions (69). Therefore, tailoring the caregiver-care receiver relationship is crucial when applying collaborative care approaches.

## 5 Limitations

This review's findings also highlight several limitations. Firstly, the use of various interrelated terms related to trust, with unclear definitions, led to interpretation challenges, as these terms can be understood differently. The authors of the articles cited may have differing interpretations, and this terminology variation might have caused some articles to be excluded. Additionally, including studies without a formal quality assessment increases the risk of incorporating research with varying levels of methodological rigor. Without assessing quality, the findings may not accurately represent the true state of the literature. For instance, including studies with methodological flaws can lead to overestimations or underestimations of effects, ultimately affecting the reliability of the conclusions drawn. Consequently, the overall conclusions of the review may combine both high-quality and low-quality evidence, potentially distorting the understanding of the topic. Secondly, the limited number of assistive technologies analyzed and the lack of significant quantitative differences among the types of assistive technologies make it challenging to draw meaningful conclusions regarding their impact on trust in long-term care. Thirdly, grey literature, including books and theses, was excluded for feasibility reasons. Focusing solely on published data may also overlook the effects of publication bias on this review, potentially limiting the comprehensiveness of the assessment. Additionally, without restricting physical conditions, we have obtained the trust dynamics in long-term care settings. Perhaps this is not applicable to every disease. Nonetheless, the insights from this review are valuable. They will assist operators of long-term care facilities in making informed decisions about technology adoption in care service strategies, ultimately enhancing the quality of long-term care.

## 6 Conclusion

This review examined long-term care involving assistive technology. As for the implications, it highlights the critical role of assistive technology in the care receiver-caregiver trust dynamic. The trust dynamic involves prompters, trustworthiness characteristics, trust in assistive technology, trust attitude, trust intention, trust behavior, evaluation, and habit. Additionally, this review summarized the factors that influence the trust dynamic; they included care receiver-related factors, caregiver-related factors, and assistive technology-related factors, which cover demographics, finance, sociability, experience, and morality etc. Furthermore, it revealed that assistive technology-related factors promote coordination between care receiver-related and caregiver-related factors, ultimately impacting the trust dynamics. Based on these findings, this review proposed pathways to encourage trust in long-term care by using assistive technology, promoting social support, providing positive emotional support, and focusing on collaborative assistance. This review provides theoretical guidance to long-term care facility operators, helps to build trust in long-term care settings, and promotes care receivers' well-being.

## Data Availability

The original contributions presented in the study are included in the article/Supplementary Material, further inquiries can be directed to the corresponding author.

## References

[1] Barber B. The Logic and Limits of Trust. New Brunswick, NJ: Rutgers University Press (1983).

[2] Mishra J Morrissey MA. Trust in employee/employer relationships: a survey of West Michigan managers. Public Pers Manage. (1990) 19(4):443–86. 10.1177/009102609001900408

[3] Ryan T Nolan M Reid D Enderby P. Using the senses framework to achieve relationship-centred dementia care services: a case example. Dementia. (2008) 7(1):71–93. 10.1177/1471301207085368

[4] Bordin ES. The generalizability of the psychoanalytic concept of the working alliance. Psychother Theory Res Pract. (1979) 16(3):252. 10.1037/h0085885

[5] Mayer RC Davis JH Schoorman FD. An integrative model of organizational trust. Acad Manag Rev. (1995) 20(3):709–34. 10.2307/258792

[6] Zhang X Zhang Q. Online trust forming mechanism: approaches and an integrated model. Proceedings of the 7th International Conference on Electronic Commerce (2005). p. 201–9. 10.1145/1089551.1089591

[7] Song H Ryan M Tendulkar S Fisher J Martin J Peters AS Team dynamics, clinical work satisfaction, and patient care coordination between primary care providers: a mixed methods study. Health Care Manage Rev. (2017) 42(1):28–41. 10.1097/HMR.0000000000000091 26545206

[8] Thorne SE Robinson CA. Reciprocal trust in health care relationships. J Adv Nurs. (1988) 13(6):782–9. 10.1111/j.1365-2648.1988.tb00570.x 3230220

[9] Vilhjálmsdóttir RA Hermannsdóttir A. Oft veltir lítil þúfa þungu hlassi: sjálfræðisréttur skjólstæðinga, vanlíðan vegna frávika og traust innan heilbrigðisþjónustu. Icel Rev Polit Adm. (2022) 18(1):120–37. 10.13177/irpa.a.2022.18.1.6

[10] Hunter PV Ward HA Puurveen G. Trust as a key measure of quality and safety after the restriction of family contact in Canadian long-term care settings during the COVID-19 pandemic. Health Policy. (2023) 128:18–27. 10.1016/j.healthpol.2022.12.009 36543694 PMC9756649

[11] Bahari G Mutambik I Almuqrin A Alharbi Z. Trust: how it affects the use of telemedicine in improving access to assistive technology to enhance healthcare services. Risk Manag Healthc Policy. (2024) 24(17):1859–73. 10.2147/rmhp.s469324 PMC11283829 39072188

[12] Ghasemzadeh R Kamali M. Assistive technology: use and service delivery. Iran Rehabil J. (2010) 8(1):54–9.

[13] World Health Organization. (2024). Available online at: https://www.who.int/news-room/fact-sheets/detail/assistive-technology (accessed August 23, 2024).

[14] Rani A Goyal V Goyal L. Assistive Technology for Home Comfort and Care. Computer Assistive Technologies for Physically and Cognitively Challenged Users. Sharjah: Bentham Science Publishers (2023). p. 73–97. 10.2174/9789815079159123020006

[15] Chinho N Choi Y Patterson P. Reducing the burdens of paid caregivers of older adults by using assistive technology: a scoping review. West J Nurs Res. (2024) 46(4):315–26. 10.1177/01939459241234233 38420931 PMC10955782

[16] Balasubramanian GV Beaney P Chambers R. Digital personal assistants are smart ways for assistive technology to aid the health and wellbeing of patients and carers. BMC Geriatr. (2021) 21:1. 10.1186/s12877-021-02436-y 34781881 PMC8591585

[17] Boccuto F Vizza P De Rosa S Tradigo G Veltri P Torella D How patients feel with telemedicine devices as an enabling factor for personalised medicine: a preliminary study. Stud Health Technol Inform. (2024) 314:168–72. 10.3233/shti240086 38785025

[18] Massardi S Pinto-Fernandez D Babič J Dežman M Trošt A Grosu V Relevance of hazards in exoskeleton applications: a survey-based enquiry. J Neuroeng Rehabil. (2023) 20(1):68. 10.1186/s12984-023-01191-y 37259115 PMC10230768

[19] Endter C Migala S Münch A Richter A. Care-ethical considerations of technology-care-assemblages. J Aging Stud. (2024) 68:101209. 10.1016/j.jaging.2024.101209 38458728

[20] Shie AJ Huang YF Li GY Lyu WY Yang M Dai YY Exploring the relationship between hospital service quality, patient trust, and loyalty from a service encounter perspective in elderly with chronic diseases. Front Public Health. (2022) 10:876266. 10.3389/fpubh.2022.876266 35692341 PMC9174694

[21] Page MJ McKenzie JE Bossuyt PM Boutron I Hoffmann TC Mulrow CD The PRISMA 2020 statement: an updated guideline for reporting systematic reviews. Br Med J. (2021) 10(89):2–10. 10.1186/s13643-021-01626-4 PMC8008539 33781348

[22] Harcourt D Rumsey N. Mastectomy patients’ decision-making for or against immediate breast reconstruction. Psycho-oncology: journal of the psychological. Soc Behav Dimens Cancer. (2004) 13(2):106–15. 10.1002/pon.711 14872529

[23] Cronin P Ryan F Coughlan M. Undertaking a literature review: a step-by-step approach. Br J Nurs. (2008) 17(1):38–43. 10.12968/bjon.2008.17.1.28059 18399395

[24] Lynn JD Rondón-Sulbarán J Quinn E Ryan A McCormack B Martin S. A systematic review of electronic assistive technology within supporting living environments for people with dementia. Dementia. (2019) 18(7-8):2371–435. 10.1177/1471301217733649 28990408

[25] Vichitvanichphong S Talaei-Khoei A Kerr D Ghapanchi AH. Assistive technologies for aged care: comparative literature survey on the effectiveness of theories for supportive and empowering technologies. Inf Technol People. (2018) 31(2):405–27. 10.1108/ITP-03-2017-0090

[26] Viera AJ Garrett JM. Understanding interobserver agreement: the kappa statistic. Fam Med. (2005) 37(5):360–3. 15883903

[27] Mishra S Laplante-Levesque A Barbareschi G Witte LD Abdi S Spann A Assistive technology needs, access and coverage, and related barriers and facilitators in the WHO European region: a scoping review. Disabil Rehabil Assist Technol. (2024) 19(2):474–85. 10.1080/17483107.2022.2099021 35906719

[28] Scholz N. Assistive Technologies to Support People with Disabilities (2015). http://epthinktank.eu/2015/06/22/assistive-technologies-to-support-people-with-disabilities/ (accessed August 23, 2024).

[29] Thomas J Harden A. Methods for the thematic synthesis of qualitative research in systematic reviews. BMC Med Res Methodol. (2008) 8:1. 10.1186/1471-2288-8-45 18616818 PMC2478656

[30] Miles MB Huberman AM Saldana J. Qualitative Data Analysis: A Methods Sourcebook. Washington, DC: SAGE Publications (2014). p. 3. 10.1016/0149-7189(86)90041-8

[31] Murphy E Doyle J Hannigan C Smith S Kuiper J Jacobs A Perceptions and use of technology to support older adults with multimorbidity. Harnessing the Power of Technology to Improve Lives. (2017). p. 160–7. 28873794

[32] Donald M Beanlands H Straus S Harwood L Herrington G Waldvogel B A research protocol for implementation and evaluation of a patient-focused eHealth intervention for chronic kidney disease. Glob Implement Res Appl. (2022) 2(1):85–94. 10.1007/s43477-022-00038-3 35402999 PMC8938369

[33] Mehrabian S Extra J Wu YH Pino M Traykov L Rigaud AS. The perceptions of cognitively impaired patients and their caregivers of a home telecare system. Med Dev Evid Res. (2014) 2015(8):21–9. 10.2147/MDER.S70520 PMC4277238 25552909

[34] Ziefle M Rocker C Holzinger A. Medical technology in smart homes: exploring the user’s perspective on privacy, intimacy and trust. 2011 IEEE 35th Annual Computer Software and Applications Conference Workshops (2011). p. 410–5. 10.1109/COMPSACW.2011.75

[35] Copolillo AE. Use of mobility devices: the decision-making process of nine African-American older adults. Occup Ther J Res. (2001) 21(3):185–200. 10.1177/153944920102100303

[36] Barker DJ Reid D Cott C. Acceptance and meanings of wheelchair use in senior stroke survivors. Am J Occup Ther. (2004) 58(2):221–30. 10.5014/ajot.58.2.221 15068158

[37] Lindqvist E Nygård L Borell L. Significant junctures on the way towards becoming a user of assistive technology in Alzheimer’s disease. Scand J Occup Ther. (2013) 20(5):386–96. 10.3109/11038128.2013.766761 23394183

[38] Gramstad A Storli SL Hamran T. Exploring the meaning of a new assistive technology device for older individuals. Disabil Rehabil Assist Technol. (2014) 9(6):493–8. 10.3109/17483107.2014.921249 24839989

[39] Southall K Gagné JP Leroux T. Factors that influence the use of assistance technologies by older adults who have a hearing loss: factores que influyen en el uso de tecnologías de asistencia en adultos mayores con hipoacusia. Int J Audiol. (2006) 45(4):252–9. 10.1080/14992020500258586 16684707

[40] Pettersson I Appelros P Ahlström G. Lifeworld perspectives utilizing assistive devices: individuals, lived experience following a stroke. Can J Occup Ther. (2007) 74(1):15–26. 10.2182/cjot.06.05 17319319

[41] Huniche L Dinesen B Nielsen C Grann O Toft E. Patients’ use of self-monitored readings for managing everyday life with COPD: a qualitative study. Telemed e-Health. (2013) 19(5):396–402. 10.1089/tmj.2012.0135 23531094

[42] Lu JF Chi MJ Chen CM. Advocacy of home telehealth care among consumers with chronic conditions. J Clin Nurs. (2014) 23(5-6):811–9. 10.1111/jocn.12156 23796027

[43] Fairbrother P Pinnock H Hanley J McCloughan L Sheikh A Pagliari C Continuity, but at what cost? The impact of telemonitoring COPD on continuities of care: a qualitative study. Prim Care Respir J. (2012) 21(3):322–8. 10.4104/pcrj.2012.00068 22875143 PMC6547965

[44] Chang CP Lee TT Mills ME. Experience of home telehealth technology in older patients with diabetes. CIN: computers, informatics. Nursing (Brux). (2017) 35(10):530–7. 10.1097/CIN.0000000000000341 28291156

[45] Fairbrother P Pinnock H Hanley J McCloughan L Sheikh A Pagliari C Exploring telemonitoring and self-management by patients with chronic obstructive pulmonary disease: a qualitative study embedded in a randomized controlled trial. Patient Educ Couns. (2013) 93(3):403–10. 10.1016/j.pec.2013.04.003 23647981

[46] Conradie P Mioch T Saldien J. Blind user requirements to support tactile mobility. Tactile Haptic User Interfaces for Tabletops and Tablets (TacTT 2014) (2014). p. 48–53

[47] Wilkowska W Ziefle M. Determinants of trust in acceptance of medical assistive technologies. Information and Communication Technologies for Ageing Well and e-Health: 4th International Conference, ICT4AWE 2018 (2018). p. 45–65. 10.1007/978-3-030-15736-4_3

[48] Harrefors C Sävenstedt S Lundquist A Lundquist B Axelsson K. Professional caregivers’ perceptions on how persons with mild dementia might experience the usage of a digital photo diary. Open Nurs J. (2012) 6(20):20–9. 10.2174/1874434601206010020 22509232 PMC3322432

[49] Sun N Rau PL. The acceptance of personal health devices among patients with chronic conditions. Int J Med Inf. (2015) 84(4):288–97. 10.1016/j.ijmedinf.2015.01.002 25655783

[50] Materia FT Smyth JM. Acceptability and concerns about innovative wearable health sensors in persons with and without chronic disease diagnosis. Internet Interv. (2024) 35:100702. 10.1016/j.invent.2023.100702 38221944 PMC10787257

[51] Wang X Wang Y. Analysis of trust factors for AI-assisted diagnosis in intelligent healthcare: personalized management strategies in chronic disease management. Expert Syst Appl. (2024) 255:124499. 10.1016/j.eswa.2024.124499

[52] Tan SH Yap YY Tan SK Wong CK. Informal caregivers’ perception of assistive robots in eldercare. Journal of open innovation: technology. Market Complex. (2024) 10(1):100234. 10.1016/j.joitmc.2024.100234

[53] Farina N Sherlock G Thomas S Lowry RG Banerjee S. Acceptability and feasibility of wearing activity monitors in community-dwelling older adults with dementia. Int J Geriatr Psychiatry. (2019) 34(4):617–24. 10.1002/gps.5064 30701592

[54] Milallos R Tibdewal V Wang Y Ogueh Udegbe A Oh T. “Would the smart cane benefit me?”: perceptions of the visually impaired towards smart canes. Proceedings of the 23rd International ACM SIGACCESS Conference on Computers and Accessibility (2021). p. 1–3. 10.1145/3441852.3476524

[55] Ventura S Ottoboni G Lullini G Chattat R Simoncini L Magni E Co-designing an interactive artificial intelligent system with post-stroke patients and caregivers to augment the lost abilities and improve their quality of life: a human-centric approach. Front Public Health. (2023) 11:1227748. 10.3389/fpubh.2023.1227748 37808976 PMC10551166

[56] Wilkowska W Gaul S Ziefle M. A small but significant difference–the role of gender on acceptance of medical assistive technologies. HCI in Work and Learning, Life and Leisure: 6th Symposium of the Workgroup Human-Computer Interaction and Usability Engineering (2010). p. 82–100. 10.1007/978-3-642-16607-5_6

[57] Lingg N Demiris Y. Building trust in assistive robotics: insights from a real-world mobile navigation experiment. Proceedings of the First International Symposium on Trustworthy Autonomous Systems (2023). p. 1–7. 10.1145/3597512.3597519

[58] Pino M Boulay M Jouen F Rigaud AS. “Are we ready for robots that care for US?” attitudes and opinions of older adults toward socially assistive robots. Front Aging Neurosci. (2015) 7:141. 10.3389/fnagi.2015.00141 26257646 PMC4512026

[59] Heerink M Kröse B Evers V Wielinga B. Assessing acceptance of assistive social agent technology by older adults: the Almere model. Int J Soc Robot. (2010) 2(4):361–75. 10.1007/s12369-010-0068-5

[60] Skymne C Dahlin-Ivanoff S Claesson L Eklund K. Getting used to assistive devices: ambivalent experiences by frail elderly persons. Scand J Occup Ther. (2012) 19(2):194–203. 10.3109/11038128.2011.569757 21534712

[61] Otten S Wilkowska W Offermann J Ziefle M. Trust in and acceptance of video-based AAL technologies. ICT4AWE (2023). p. 126–34. 10.5220/0011785500003476 PMC9921302 36772195

[62] Mikesell L Bontempo AC. Healthcare providers’ impact on the care experiences of patients with endometriosis: the value of trust. Health Commun. (2023) 38(10):1981–93. 10.1080/10410236.2022.2048468 35287508

[63] Olek D Uchmanowicz I Chudiak A Jankowska-Polańska B. Wpływ akceptacji choroby na jakość życia chorych w przewlekłej obturacyjnej chorobie płuc. Probl Pielęgniarstwa. (2014) 22(4):471–6. 10.5603/ARM.45552

[64] Thordardottir B Malmgren Fänge A Lethin C Rodriguez Gatta D Chiatti C. Acceptance and use of innovative assistive technologies among people with cognitive impairment and their caregivers: a systematic review. BioMed Res Int. (2019) 1:9196729. 10.1155/2019/919672966 PMC6431399 30956989

[65] Cahn A Akirov A Raz I. Digital health technology and diabetes management. J Diabetes. (2018) 10(1):10–7. 10.1111/1753-0407.12606 28872765

[66] Sqalli MT Al-Thani D Qaraqe M Fernandez-Luque L. Perspectives on Human-AI Interaction Applied to Health and Wellness Management: Between Milestones and Hurdles. Multiple Perspectives on Artificial Intelligence in Healthcare: Opportunities and Challenges. Cham: Springer (2021). p. 41–51. 10.1007/978-3-030-67303-1_4

[67] Eicher C Kiselev J Brukamp K Kiemel D Spittel S Maier A Experiences with assistive technologies and devices (ATD) in patients with amyotrophic lateral sclerosis (ALS) and their caregivers. Technol Disabil. (2019) 31(4):203–15. 10.3233/TAD-190227

[68] Wasson JH Johnson DJ Benjamin R Phillips J MacKenzie TA. Patients report positive impacts of collaborative care. J Ambul Care Manage. (2006) 29(3):199–206. 10.1097/00004479-200607000-00004 16788352

[69] Waterworth S Luker KA. Reluctant collaborators: do patients want to be involved in decisions concerning care? J Adv Nurs. (1990) 15(8):971–6. 10.1111/j.1365-2648.1990.tb01953.x 2229694

